# 

**Published:** 1873-07

**Authors:** 


					﻿Books.
For want of space, we are unable to notice the books and pam-
phlets sent us in this number.
Payments received during the past month for subscriptions to
the Southern Medical Record will be acknowledged in our
next.
(Uie Southern 4Hedicnf Record.
EDITED BY
T. S. POWELL, M.D.,
AN D
W. T. OOLDSMITH, M. D.
ASSOCIATE EDITORS:
GEORGIA.	ALABAMA.	VIRGINIA.
Robert Battey, M.D.	J. C. Knox, M.D.	Charles S. Lewis, M.D.
R. C. Word, M.D.	Geo. F. Taylor, M.D.	John IL Claiborne. M.D.
W. L. Kirkpatrick, M.D.	J. B. Barnett, M.D.	F. Horner, Jr., M.D.
James A. Long, M.D.	S.	M.	Hogan, M.D.	R. J. Preston, M.D,
W. L. M. Harris, M.D.	D.	S.	Hopping, M.D.	A. S. I’ayne. M.D.
H. L Battle, M.D.	J.	T.	Searcy. M.D.	J. E. Lockridge. M.D.
W. C. Musgrove, MD	S.	F.	Hill, M.D.	J. S. Wharton, M.D.	.
L B. Boucnelle, M.D.	Wm. O. Owen, M.D.
John C. Drake, M.D.	MISSISSIPPI,	Sam’l P. Ripley. M.D.
E. F. Knott, M.D.	Benj. Blacklord.M.D.
Thomas Burdell, M.D.	C. B. Gallaway, M.D,	E. A. Drewry, M.D.
E. H. W. Hunger, M.D.	Edward Lea. M.D.	Rob. B. Tunstall, M.D.
V.	S. Hitt, M.D.	S. V. I). Hill, M.D.	A.	J.	Terrell. M.D.
J. E. Pope, M.D.	W. B. Harvey, M.D.	W.	F. Barr. M.D.
J. A. Hunnicutt, M.D.	J. W. M. Shattuck, M.D.	L. B. Anderson, M.D.
A. H. Brantley, M.D.	E. T. Henry, M.D.	S.	L.	Jepson, M.D., W. Va.
J. J. Knott. M.D.	T. P. Lockwood, M.D.	J.	M	Lazzell. M.D.
J. C. Wisdom, M.D.	Robert Kells, M.D.
W.	W. Leake. M.D.
SOUTH CAROLINA.	TENNESSEE.	TEXAS.
E. T. McSwain, M.D?	J. D. Tucker, M.D.	S. M. Welch, M.D.
G. M. Rivers, M.D.	Swan M. Burnett, M.D.	T. J. Heard. M.D.
W. A. Heard. M.D.
A. R. Kilpatrick. M.D,
NORTH CAROLINA.
ARKANSAS.	J.	R. Hughes, M.D.	E. A. Anderson, M.D.
S. S. Satchwell, M.D.	II. T. Bahnson, M.D.
A. D. Hodge, M.D.	A.	B. Pierce, M.D.	Willis Alston, M.D.
J. K. Hodge. M.D.	H.	Kelly. M.D.	Thos. F. Wood, M.D.
Geo. A. Foote, M.D.	N. J. Pittman, M.D.
CORRESPONDING EDITORS:
Ely Van DeWarker, M. D., New York.	M. II. Henry. M.D.. New York,
John H. Weir, M.D., Hlinois.	Surgeon to New York Dispensary, Department
Thomas J Gallaher, M. D, Pennsylvania.	of Venerial and skin Dieeases.
Robert Robson, M.D., Indiana.	Wm. R. Whitehead, M.D., Colorado Territory.
C. C. F. Gay, M.D., New York.	A. Patton. M.D.. Indiana.
Surgeon of Buffalo General Hospital.	Benjamin Howard, M.D., New York.
W. T Taylor, M.D , Kentucky.	Chas Kearns, M D., Kentucky.
J. Orne Green, Boston, Maesachusets.	S. C. Busey, M.D., Washington, D. C.
B. W. Stone, M.D., Kentucky,	A. W. Hobson, M.D., Arkansas.
Assistant Physician Western Lunatic Asylum. A. W. Calhoun. M.D., now of Berlin. Prussia.
James Tyson, M.D., Philadelphia, Pa. I	T. Curtis Smith, Ohio.
W. W. Leen, M.D., Philadelphia.	Geo. Strawbridge, M.D., Philadelphia.
All Business Letters should be addressed to POWELL GOLDSMITH,
Post Office Box 527, Atlanta, Georgia.
				

